# Tumoral and Peritumoral Radiomics for Preoperative Prediction of Visceral Pleural Invasion in Lung Adenocarcinoma

**DOI:** 10.3390/cancers17244001

**Published:** 2025-12-16

**Authors:** Filippo Tommaso Gallina, Sonia Lucchese, Antonello Vidiri, Francesca Laganaro, Sergio Ruggiero, Doriana Vergara, Riccardo Tajè, Edoardo Mercadante, Paolo Visca, Simona Marzi

**Affiliations:** 1 Thoracic Surgery Unit, IRCCS Regina Elena National Cancer Institute, 00144 Rome, Italy; filippo.gallina@ifo.it (F.T.G.); r.taje@virgilio.it (R.T.); edoardo.mercadante@ifo.it (E.M.); 2 Radiology Unit IRCCS Regina Elena National Cancer Institute, Via Elio Chianesi 53, 00144 Rome, Italy; sonia.lucchese@ifo.it (S.L.); antonello.vidiri@ifo.it (A.V.); sergio.ruggiero@ifo.it (S.R.); doriana.vergara@ifo.it (D.V.); 3 Pathology Unit IRCCS Regina Elena National Cancer Institute, Via Elio Chianesi 53, 00144 Rome, Italy; paolo.visca@ifo.it; 4 Medical Physics Unit, IRCCS Regina Elena National Cancer Institute, Via Elio Chianesi 53, 00144 Rome, Italy; simona.marzi@ifo.it

**Keywords:** lung adenocarcinoma, visceral pleural invasion, computed tomography, radiomics, prediction

## Abstract

Background:The presence of visceral pleural invasion (VPI) is associated with increased risk of recurrence and reduced overall survival following surgical resection. We aimed to develop machine learning (ML)-based classification models that integrate clinical variables and both tumoral and peritumoral radiomic features to predict VPI in patients with lung adenocarcinoma before surgery. Methods: We retrospectively enrolled 118 patients, including 80 (68%) without VPI and 38 (32%) with histologically confirmed VPI. All patients underwent preoperative contrast-enhanced CT scans. Tumor volumes were manually segmented, and isotropic expansions of 3, 5, and 10 mm were automatically generated to define peritumoral regions. The dataset was randomly split into training (70%) and validation (30%) cohorts. Radiomic features and clinical data were used to train multiple ML algorithms. Results: Pleural Tag Sign and the Worst Histotype were identified as the strongest clinical predictors of VPI. The combined model, integrating radiomics from the lesion and clinical variables, achieved the highest training accuracy of 0.88 (95% CI: 0.80–0.92) and validation accuracy of 0.83 (95% CI: 0.68–0.92). Conclusions: VPI is associated with detectable alterations in both tumoral and peritumoral microenvironment on contrast-enhanced CT. Incorporating radiomic features with clinical data enabled improved model performance compared to clinical-only models, yielding very good accuracies. This approach may support surgical planning and patient risk stratification. Further prospective studies are needed to validate these findings and assess their clinical impact.

## Simple Summary

Visceral pleural invasion (VPI) is a negative prognostic factor in non-small cell lung cancer (NSCLC). This study aimed to build machine learning (ML) models that combine clinical data with tumoral and peritumoral radiomic features to predict VPI in lung adenocarcinoma preoperatively. A retrospective analysis was conducted on 118 patients—80 (68%) without and 38 (32%) with histologically confirmed VPI—who underwent contrast-enhanced CT before surgery. Tumors were manually segmented, and peritumoral regions were generated through isotropic expansions of 3, 5, and 10 mm. Multiple ML classifiers were trained using both clinical and radiomic features. Among clinical variables, the Pleural Tag Sign and Worst Histotype were the most relevant predictors. The integrated radiomic-clinical model achieved accuracies of 0.88 in the training and 0.83 in the validation cohorts. These findings suggest that combining radiomics with clinical parameters can improve preoperative VPI prediction, supporting surgical decision-making and risk stratification.

## 1. Introduction

Lung cancer remains one of the most prevalent and lethal malignancies worldwide, contributing to cancer-related morbidity and mortality, with non-small cell lung cancer (NSCLC) accounting for 80–85% of cases. Within NSCLC, lung adenocarcinoma (LA) is the predominant histological subtype, particularly among non-smokers and individuals with peripheral lung lesions [1,2]. Visceral pleural invasion (VPI), defined as tumor extension beyond the elastic layer of the visceral pleura, is an important prognostic factor, particularly in early-stage lung adenocarcinoma. Its presence is associated with a higher risk of recurrence and reduced overall survival after surgical resection, and it leads to upstaging from T1a–c to T2a, thereby advancing the overall stage from Ia to Ib according to the 8th edition of the Union for International Cancer Control/American Joint Committee on Cancer (UICC/AJCC) TNM staging system [3]. Thus, the identification of VPI status before surgery plays a pivotal role in thoracic surgical decision-making, influencing the extent of resection, preferring a lobectomy instead of sublobectomy [4], and the consideration of systemic treatment escalation.

Moreover, the introduction of immunotherapy—particularly immune checkpoint inhibitors—is progressively modifying current treatment algorithms in NSCLC, with growing evidence supporting its potential use even in the neoadjuvant setting for early-stage tumors [5].

Several studies have already investigated the role of computed tomography (CT) in predicting the presence of VPI [6,7,8,9,10]. For instance, tumors presenting as ground-glass nodules or lacking pleural contact are associated with a low likelihood of VPI [8,11]. In contrast, CT findings such as direct tumor–pleural contact, pleural indentation, and pleural tags are recognized as high-risk indicators of VPI [6,7,12]. However, despite these associations, the predictive accuracy of VPI based solely on morphological CT signs remains limited.

Recent advances in radiomics have enabled the extraction of high-dimensional quantitative features from medical images, offering a more nuanced characterization of tumour phenotype and microenvironment. Recent studies [8,9,10,13,14,15] have shown that incorporating radiomic features—particularly those derived from both intratumoral and peritumoral regions—can improve the accuracy of predictive models for assessing pathological invasiveness in lung cancer. Nonetheless, most existing radiomics-based models for VPI prediction have primarily focused on intratumoral features or on combined intratumoral and peritumoral regions, while less attention has been devoted to the peritumoral region alone. This area, however, may contain valuable diagnostic information, allowing the detection of early alterations at the tumor–pleura interface that are not otherwise appreciable on conventional CT imaging.

In the present study, we aim to develop and validate machine learning (ML)-based classification models that integrate clinical variables with tumoral and peritumoral radiomic features to predict VPI in patients with lung adenocarcinoma preoperatively.

## 2. Materials and Methods

### 2.1. Patient Population

This single-center retrospective study included patients with clinical node-negative early-stage NSCLC treated at Regina Elena National Cancer Institute, who underwent radical resection between January 2021 and December 2023 with available preoperative CT scans. The study was approved by the institutional ethics committee (RS1832/23), and informed consent was waived due to the retrospective nature of the study.

The preoperative workup consisted of brain, chest, and upper abdominal computed tomography (CT) scans, along with F18-fluorodeoxyglucose positron emission tomography (FDG-PET), to rule out the presence of multiple pulmonary nodules, as well as hepatic, adrenal, or cerebral metastases, and to assess hilar and mediastinal lymph node involvement. When indicated, nodal disease was further evaluated by endoscopic or endobronchial ultrasound-guided fine-needle aspiration, performed according to current guidelines. Following surgical resection, all formalin-fixed paraffin-embedded (FFPE) specimens were examined by dedicated pathologists for confirmation of the diagnosis and evaluation of tumor cellularity. The predominant invasive histologic subtype of lung adenocarcinoma was categorized as acinar, cribriform, papillary, micropapillary, solid, clear-cell, lepidic, mixed, mucinous, pleomorphic, or undifferentiated. The evaluation of VPI was performed on hematoxylin–eosin (H&E) stained slides and, when necessary, with elastic fiber staining (e.g., elastic Van Gieson) to identify tumor extension beyond the elastic layer of the visceral pleura. VPI was classified according to the current IASLC/WHO recommendations: PL0, tumor within the lung parenchyma or pleura but not beyond the elastic layer; PL1, invasion beyond the elastic layer; PL2, invasion to the pleural surface; and PL3, invasion of the parietal pleura, chest wall, or other adjacent structures. For this study, PL1 and PL2 were considered positive for visceral pleural invasion (VPI+), whereas PL0 was considered negative (VPI−). Cases with PL3 involvement were excluded. We included patients with clinical stage I–III NSCLC who had undergone complete preoperative staging according to current guidelines, received radical surgical treatment following IASLC recommendations, and had available preoperative contrast-enhanced CT scans. Patients were excluded if they had clinical stage IV disease, clinical N2 involvement, or had received preoperative chemotherapy or radiotherapy.

### 2.2. Imaging Protocol

CT acquisition was performed using multidetector-row CT scanners from three different manufacturers (Incisive CT, Philips Medical Systems, Best, The Netherlands; iCT SP, Philips Medical Systems, Best, The Netherlands; Optima CT660, GE Medical Systems, Waukesha, WI, USA). All examinations were conducted with patients in the supine position during full inspiration, covering the entire thoracic region. The CT acquisition parameters were as follows: tube voltage, 120 kV; tube current, 120–250 mA; reconstruction slice thickness, 0.75 mm or 1.25 mm; matrix size, 512 × 512; and field of view, 500 mm.

All images were subsequently reviewed using lung window settings (window width: 1500 HU; window level: −650 HU). Pulmonary nodules were categorized as solid nodules (including part-solid nodules with a solid component exceeding 80%), sub-solid nodules (ground-glass nodules that contain one or more solid components less than 80%), or ground-glass nodules (circumscribed areas of increased lung attenuation with preservation of bronchial and vascular margins without solid parts in the context). Furthermore, nodules were classified based on their relationship to the pleura into two categories: (a) pleural-attached nodules, defined as those directly abutting the pleural surface or connected to it via at least one linear pleural tag; and (b) non-pleural-attached nodules, with no contact with the pleura on any CT slice. Additionally, nodules were assessed for the presence or absence of spiculation.

The mean interval between the diagnostic CT study and surgery was 30.6 ± 19.9 days.

### 2.3. Delineation of Volumes of Interest

For each patient, four distinct volumes of interest (VOIs) were analyzed: the lung lesion and its peritumoral regions obtained by expanding the lesion margins by 3 mm, 5 mm, and 10 mm. The lesion was manually segmented slice by slice by an expert radiologist using semi-automatic tools in the 3D Slicer software (version 5.8.1) [16].

To generate the peritumoral regions, the entire lung volume was first automatically segmented using the Lung CT Segmenter extension in 3D Slicer software. Peritumoral VOIs were then generated using a custom MATLAB script (Release R2021b, MathWorks Inc., Natick, MA, USA). First, an isotropic kernel was applied to each manually segmented lesion to create isotropic expansions of 3 mm, 5 mm, and 10 mm around the tumor. Then, to ensure that the expanded volumes remained confined to the lung parenchyma, each expansion was intersected with the previously segmented lung mask. Finally, the lesion volume was subtracted from each expanded VOI to isolate only the peritumoral tissue.

**Figure 1:**
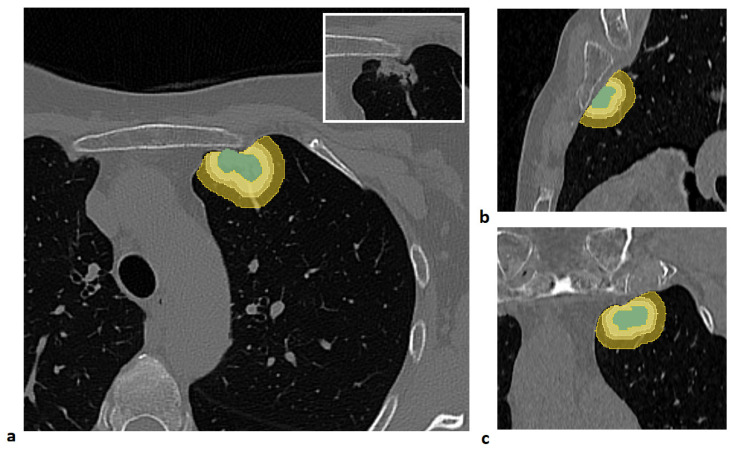
Example of lesion segmentation and the corresponding peritumoral regions generated by isotropic expansions of 3 mm, 5 mm, and 10 mm, constrained within the lung parenchyma mask, shown in the axial (**a**), sagittal (**b**), and coronal (**c**) views. The lesion is shown in the box at the top right of panel (**a**).

An example of a segmented lesion and the corresponding peritumoral regions is shown in Figure 1.

### 2.4. Feature Extraction

Radiomic features were extracted from each VOI (i.e., the entire lesion and its peritumoral regions at 3 mm, 5 mm, and 10 mm) using the open-source LIFEx software (version 7.7.0) [17], in compliance with the Image Biomarker Standardization Initiative (IBSI) guidelines [18].

Prior to feature extraction, each volume was resampled to an isotropic voxel size of 1 mm and discretized using a fixed bin width of 25. A total of 119 radiomic features were extracted for each structure, spanning the following categories: morphological, first-order statistics (intensity-based and intensity histogram), Grey-Level Co-occurrence Matrix (GLCM), Grey-Level Run Length Matrix (GLRLM), Neighborhood Grey Tone Difference Matrix (NGTDM), and Grey-Level Size Zone Matrix (GLSZM) [18]. A comprehensive list of the extracted features is available in Supplementary Table S1.

To reduce scanner-related variability across the four acquisition systems, radiomic features were harmonized using the ComBat algorithm [19], implemented through the neuroCombat package (version 1.0.14) in RStudio software (version 2024.12.0; Posit Software, PBC, Boston, MA, USA).

### 2.5. ML Modelling

We developed various predictive models: a clinical model based on clinical variables and CT-based morphological features, i.e., manually assessed morphological features reported by radiologists in the CT reports (such as the Pleural Tag Sign or tumor size); four radiomic models, each using features extracted from a specific VOI, i.e., the entire lesion, and its peritumoral regions expanded by 3 mm, 5 mm, and 10 mm; and four combined models, each merging the most relevant clinical features with radiomic features from one of the respective VOIs, to evaluate the added value of integrating radiomic and clinical data.

Prior to modelling, all numerical features underwent a standardized preprocessing pipeline including outlier removal, Z-score normalization, and replacement of missing values with the median. Features highly correlated with lesion volume (Spearman’s Rho ≥ 0.8) were normalized to lesion volume to mitigate size-dependent biases.

To identify the most informative variables, an ML-based feature selection process was conducted using the Adaptive Boosting (AdaBoost) classification algorithm. Features were ranked according to their importance scores, and to limit overfitting due to the small sample size, only the top 10 features were retained. Among these, the optimal subset of three predictors was selected to build the final classification models.

The dataset was randomly split into a training cohort (70%) and a validation cohort (30%), ensuring balanced class distribution through stratified splitting.

**Figure 2:**
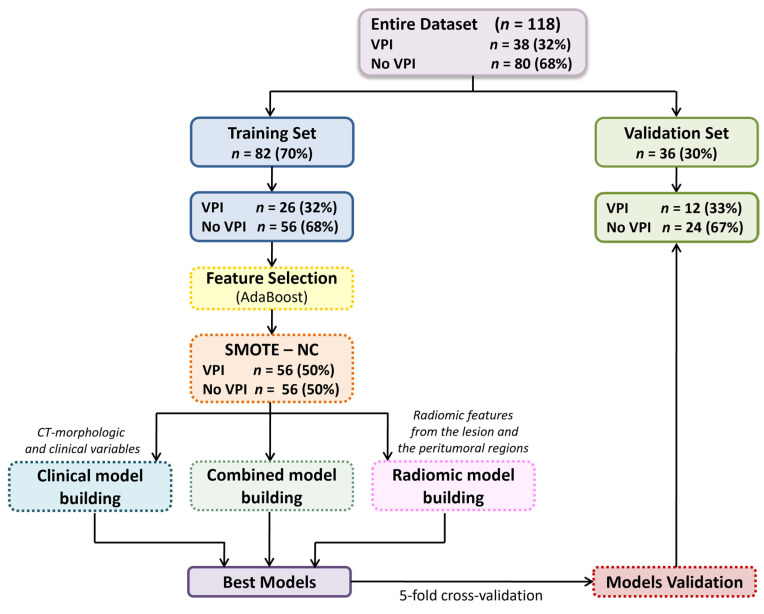
Overview of the analysis pipeline to obtain the final models. Abbreviations: VPI, Visceral Pleural Invasion; AdaBoost, Adaptive Boosting; SMOTE–NC, Synthetic Minority Over-sampling Technique for Nominal and Continuous variables.

To address class imbalance, synthetic minority class samples were generated using the Synthetic Minority Over-sampling Technique (SMOTE) [20], implemented via the RSBID (version 0.0.2.0000) package in RStudio software. The study analysis pipeline is shown in Figure 2.

A comparative evaluation of several classification algorithms was performed, including Decision Trees, Discriminant Analysis, Logistic Regression, Naive Bayes, Support Vector Machines, K-Nearest Neighbours, and Ensemble Classifiers. Hyperparameter tuning and a stratified five-fold cross-validation were applied to optimize model performance and reduce overfitting. Model performance was assessed in terms of accuracy, sensitivity, specificity, and area under the receiver operating characteristic curve (AUC). AUC values were estimated with 95% confidence intervals by bootstrapping (1000 replicates). Pairwise comparisons of predictive accuracy were performed using the mid-p McNemar test. All analyses and model development were carried out using the Statistics and Machine Learning Toolbox in MATLAB (Release R2021b).

### 2.6. Assessment of Feature Robustness to Segmentation Variability

To evaluate the sensitivity of radiomic features to potential inter-observer variability, we performed a perturbation-based robustness analysis [21]. A subset of 20 patients was selected such that the distribution of tumor sizes (median and range) closely matched that of the entire cohort. The original manual segmentations—generated by a single radiologist—were subjected to controlled geometric modifications designed to mimic plausible variations in lesion contours. These perturbations included slight erosions and dilations of the masks (2 mm) and in-plane rotations (±10°). An example of the perturbation strategies applied to the manual segmentations of a representative patient is illustrated in Supplementary Figure S1. For each perturbed segmentation, the three peritumoral regions (3, 5, and 10 mm) were re-generated following the same procedure used for the original contours, and radiomic features were re-extracted.

Feature robustness was quantified by computing intraclass correlation coefficients (ICC; two-way random-effects model, absolute agreement) between features derived from the original and perturbed masks using the Irr package (version 0.84.1) in RStudio software. Features with ICC ≥ 0.8 were considered stable with respect to segmentation variability [10]. ICC results were used a posteriori to verify that the features included in the final predictive models belonged to this robust subset, ensuring that model interpretation relied predominantly on stability-proven radiomic descriptors.

### 2.7. Model Performance Evaluation According to the Tumor Size

To assess the discriminative performance of the predictive models across different tumor dimensions, patients were stratified into three subgroups based on the tumor’s major axis: (1) ≤3 cm, (2) >3 cm and ≤5 cm, and (3) >5 cm. Model predictions were compared against the reference VPI status to generate confusion matrices and calculate performance metrics—namely, accuracy, sensitivity, and specificity—within each subgroup, for each of the proposed models.

### 2.8. Statistics

To assess the individual association of each feature with the clinical outcome, univariate statistical tests were applied. Continuous variables—including both radiomic features and continuous clinical variables—were analyzed using the Mann–Whitney U test, while categorical variables were assessed using the Chi-square test.

To minimize feature redundancy, pairwise associations among the selected variables were evaluated using the Spearman correlation coefficient for continuous variables, the Chi-square test for categorical variables, and the Kruskal–Wallis test for comparisons involving mixed data types. In cases of strong correlation between two features (Spearman’s Rho > 0.80), the variable showing the strongest univariate association with the clinical outcome was retained.

A *p*-value less than 0.05 was considered statistically significant. All statistical analyses were performed in MATLAB (Release R2021b).

## 3. Results

The study cohort consisted of 118 patients, including 80 (68%) without VPI and 38 (32%) with histologically confirmed VPI. The dataset was randomly split into a training cohort (*n* = 82) and a validation cohort (*n* = 36) using a stratified procedure to preserve class proportions. Table 1 reports the clinical characteristics of the overall population and each subset. No statistically significant differences were observed between the training and validation sets for any of the variables considered.

### 3.1. Performance of ML Models

A comprehensive summary of the models, the selected features, and their performance metrics is provided in Supplementary Table S2. All radiomic features retained in the proposed predictive models showed high robustness to segmentation variability, with ICC values > 0.8, as detailed in Supplementary Table S3. Among all the models developed, the highest predictive performance was observed in three cases: the radiomic model based on features from the lesion, the radiomic model based on the 10 mm-peritumoral region, and the combined model integrating radiomic features from the lesion volume with clinical variables.

For the radiomic model based on the lesion, the top three predictors were Integrated Intensity, Grey-Level Non-Uniformity from the GLSZM, and Root Mean Square (RMS) Intensity. For the 10 mm-peritumoral model, the most relevant features were Flatness, Intensity Skewness, and Run Variance from the GLRLM. The clinical model identified the Pleural Tag Sign and the Worst Histotype as the strongest predictors of VPI. In the combined model based on the clinical features and the radiomic predictors extracted from the lesion volume, RMS Intensity, Worst Histotype, and Pleural Tag Sign emerged as the most important variables.

Descriptive statistics for these predictors, stratified by VPI status, are reported in Table 2.

**Figure 3:**
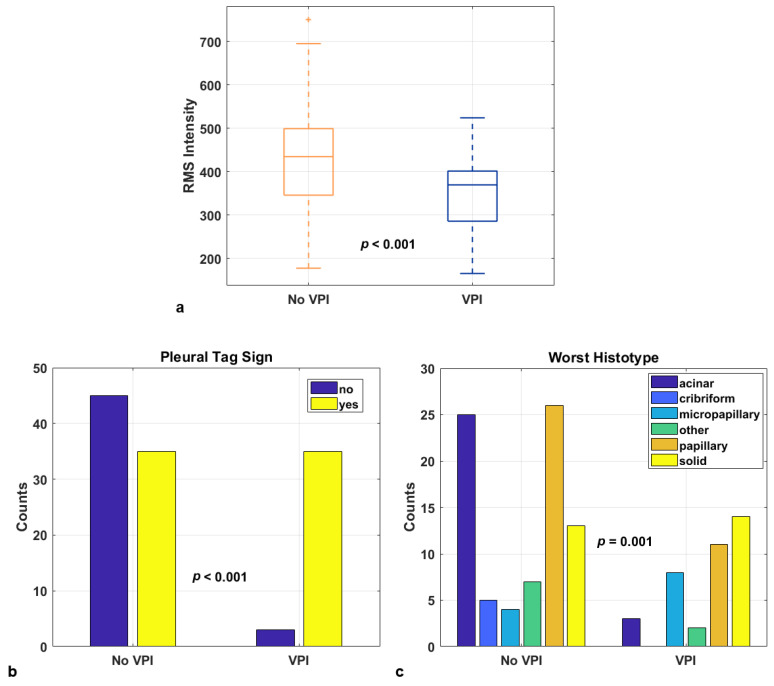
Boxplot and bar plots of the three predictors in the best-performing model for visceral pleural invasion (VPI), combining intratumoral radiomic features with clinical data: Root Mean Square (RMS) Intensity derived from the radiomic analysis of the lesion (**a**); presence of a pleural tag (**b**); and worst histotype (**c**).

Notably, although not retained in the final models, the lesion major axis length showed a significant univariate association with VPI (*p* < 0.001), while density exhibited a trend toward significance (*p* = 0.055), consistent with their recognized role in pleural invasiveness. Figure 3 shows the distribution of the top three predictors from the combined model.

Table 3 summarizes the performance of the best-performing prediction models in the training and validation sets.

The clinical model achieved an accuracy of 0.81 (95% CI: 0.73–0.87) in the training set and 0.81 (95% CI: 0.65–0.90) in the validation set. The radiomic model based on the lesion reached an accuracy of 0.80 (95% CI: 0.79–0.82) in training and 0.81 (95% CI: 0.75–0.83) in validation cohorts. The 10 mm-peritumoral radiomic model achieved an accuracy of 0.83 (95% CI: 0.75–0.89) in training and 0.81 (95% CI: 0.65–0.90) in validation. The combined model—integrating the radiomic features from the lesion with clinical data—yielded the highest AUC, with an accuracy of 0.88 (95% CI: 0.80–0.92) and 0.83 (95% CI: 0.68–0.92) in the training and validation sets, respectively.

Pairwise McNemar’s tests revealed comparable performance among the proposed models in both the training and validation sets, with the only significant difference observed in the training set between the clinical and combined models (*p* = 0.008; Supplementary Table S4).

**Figure 4:**
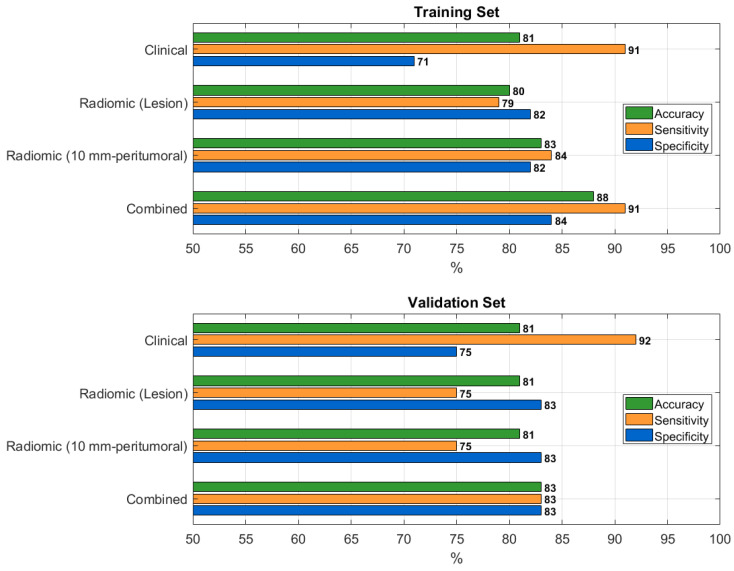
Bar plots comparing the performance metrics of the four best-performing predictive models for visceral pleural invasion in the training and validation sets.

A graphical overview comparing the performance of the four best-performing models is provided in Figure 4.

### 3.2. Model Performance by Tumor Size

In the training set, 57 out of 82 patients (70%) had a tumor size ≤ 3 cm, 19 patients (23%) had tumors between 3 and 5 cm, and six patients (7%) had tumors > 5 cm. In the validation set, 24 out of 36 patients (67%) had tumors ≤ 3 cm, nine patients (25%) between 3 and 5 cm, and three patients (8%) > 5 cm. The classification performance of the proposed models within each tumor size subgroup is summarized in Supplementary Figures S1–S5.

In the training set, the accuracy of the models ranged from 68% to 82% for tumors ≤ 3 cm and from 58% to 89% for tumors between 3 and 5 cm, depending on the model. For tumors > 5 cm, all models achieved 100% accuracy. Similarly, in the validation set, the accuracy of the models ranged from 75% to 83% for tumors ≤ 3 cm and from 56% to 89% for those between 3 and 5 cm; again, all models achieved 100% accuracy for tumors > 5 cm. Remarkably, in the validation set, the sensitivity of most models decreased in the subgroup with tumors ≤ 3 cm compared to the 3–5 cm group, except for the radiomic and combined models derived from the 3 mm peritumoral region and the combined model based on the lesion.

**Figure 5:**
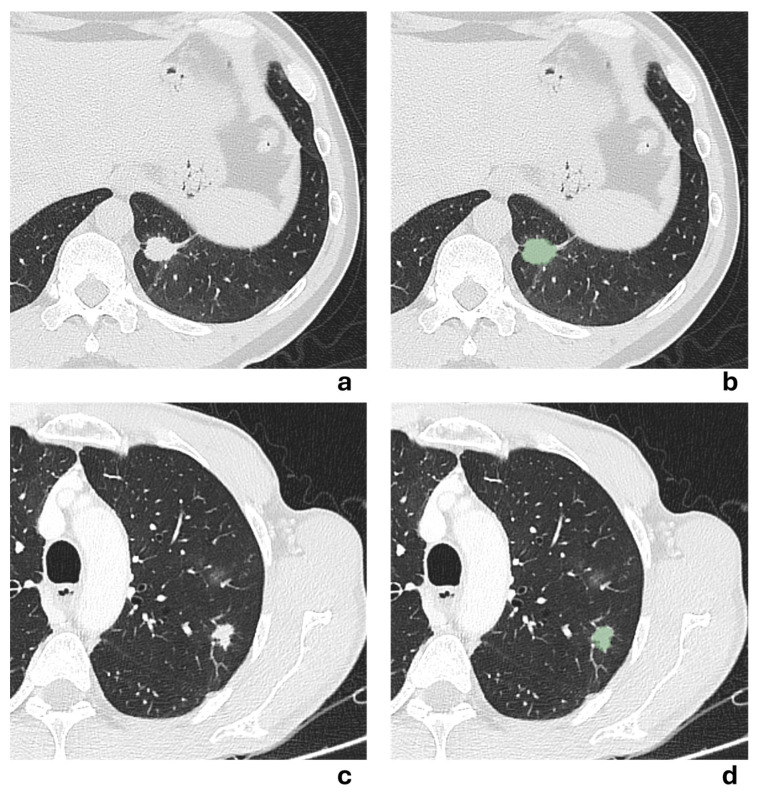
(**a**,**b**) Axial chest CT scan in lung window of a 54-year-old man showing a solid irregular nodule in the anteromedial basal segment of the left lower lobe, with evidence of a pleural tag sign, measuring 14.4 mm in major axis. The corresponding segmented lesion is shown in (**b**). Despite radiological suspicion, histological examination revealed no pleural invasion. (**c**,**d**) Axial chest CT scan in lung window of a 65-year-old man showing a solid irregular nodule in the apical segment of the left lower lobe, without evidence of pleural tag sign, measuring 22.3 mm in major axis. The corresponding segmented lesion is shown in (**d**). Histological examination revealed pleural invasion, despite no radiological evidence being suspected.

Two representative NSCLCS cases with a tumor major axis < 3 cm are illustrated in Figure 5. In the first case (a,b), the presence of a pleural tag suggested a radiological suspicion of pleural invasion, but it was not confirmed by histological examination. In the second case (c,d), no radiological suspicion of pleural invasion was raised due to the absence of a pleural tag, but histological examination revealed pleural invasion. All models failed to correctly predict the presence of pleural invasion, except for the 3 mm-peritumoral radiomic model.

## 4. Discussion

In the present study, we developed ML-based classification models that integrated clinical variables with radiomic features extracted from intratumoral lesion volume and peritumoral rims of different sizes to predict VPI in patients with lung adenocarcinoma preoperatively. Several studies have already demonstrated promising results with predictive models combining clinical variables with radiomic features [8,9,10,12,22]. Accordingly, we observed a statistically significant improvement of the combined model over the clinical model in the training set, corroborated by pairwise testing.

Beyond radiomics, previous studies have highlighted the predictive value of clinico-radiological signs for VPI prediction. For instance, Zha et al. [12] and Huang et al. [22] showed that incorporating variables such as pleural tag or pleural indentation markedly improves model performance. The pleural tag—defined as pleural traction with connecting strands—has consistently emerged as an independent predictor [12,22,23], emphasizing the relevance of tumor–pleura interactions even in conventional imaging. In our clinical model, Pleural Tag Sign and Worst Histotype were identified as significant predictors of VPI. Pleural Tag Signs were more frequently observed in patients with VPI, reflecting pleural retraction or tethering caused by tumor–pleura interaction. Furthermore, patients with VPI exhibited a lower prevalence of the acinar pattern and a higher prevalence of the solid pattern as the worst histotype, in line with the recognized association between solid morphology and more aggressive biological behavior.

Our results also support previous observations that larger tumors are associated with higher VPI risk [6,7,8,9,13,14,22]. In our cohort, the major axis length was significantly greater in VPI-positive patients; however, it was not retained in the final models, likely because pleural tag sign and histotype captured more specific, non-overlapping information among the most relevant predictors. Density also showed a trend toward significance in predicting VPI, consistent with prior reports [8,13,14,22], but was excluded due to its high correlation with histotype, which had a stronger association with VPI and effectively incorporated its predictive contribution.

Compared with purely clinical studies [7,23], which relied solely on semantic signs, radiomics-based models demonstrated an advantage in terms of accuracy. Our findings, in conjunction with evidence from literature, underscore the added value of radiomics, which can capture imaging-derived markers of heterogeneity beyond conventional predictors. In our study, VPI-positive lesions exhibited higher intratumoral Integrated Intensity, reflecting greater tumor size and density, as well as higher Grey-Level Non-Uniformity, indicating increased heterogeneity likely related to variations in cellularity, necrosis, or stromal composition. RMS Intensity was lower in VPI cases, suggesting a predominance of low-intensity voxels or fewer high-intensity voxels and a more irregular internal architecture. When considering the 10 mm-peritumoral rim, VPI-positive patients showed lower Flatness, consistent with more irregular peritumoral geometry; lower positive Skewness, reflecting fewer localized areas of low intensity with an increased proportion of intermediate-to-high intensity voxels, indicating a more homogeneous distribution of intensity values, possibly due to stromal remodeling; and lower Run Variance, suggesting reduced textural variability and a less complex peritumoral microenvironment.

We want to emphasize that our analysis was conducted using a standardized processing pipeline, including ComBat harmonization to reduce scanner variability, SMOTE oversampling to address class imbalance, and AdaBoost-based feature selection. In addition, a perturbation-based evaluation of feature robustness was performed to verify that the radiomic features contributing to the final models remained stable under simulated contour variations. Unlike previous literature that integrated intratumoral and peritumoral features [13,14], our approach focused specifically on multiple concentric peritumoral regions after excluding the tumor itself, thereby isolating the specific contribution of the surrounding tissue and providing a more rigorous assessment of the value of the peritumoral area. Moreover, evaluating three different distances enabled a data-driven comparison across spatial scales, without any a priori assumption about which rim would be most informative for VPI prediction. In this context, peritumoral features may be interpreted as quantitative surrogates of tumor and pleural microenvironment alterations, preceding the histological confirmation of VPI. The use of 3, 5, and 10 mm peritumoral rims is biologically grounded, as microscopic aerogenous spread in stage I lung adenocarcinoma is almost entirely confined within 0.3–10.5 mm of the tumor edge [24].

The relevance of peritumoral environment analysis has been previously highlighted for different purposes in NSCLC. For example, Chang et al. [25] and Vaidya et al. [26] investigated peritumoral radiomics to evaluate chemotherapy response, remarking that the peritumoral microenvironment may provide complementary prognostic information beyond intratumoral descriptors. Very recent studies have examined its role in VPI prediction. Among them, Zuo et al. [14] and Wang et al. [13] demonstrated that the inclusion of features extracted from the peritumoral margin provides complementary information, resulting in an increase in AUC compared to intratumoral models alone. Our results confirm the added predictive value of peritumoral tissue, with the 10 mm-peritumoral model achieving overall performance comparable to, and even higher in accuracy and sensitivity than, those reported by Zuo et al. [14] and Wang et al. [13].

Moreover, considering the critical role of lesion size in VPI, we assessed the discriminative performance of the predictive models across different tumor dimensions. Most models showed a reduction in sensitivity in the subgroup with tumors ≤ 3 cm, compared to the 3–5 cm group. This finding is consistent with the observations of Ahn et al. [6], who reported that VPI in small nodules occurs almost exclusively in those with a predominant solid component. Similarly, Sun et al. [23] identified the so-called jellyfish sign as a highly predictive CT feature in solid tumors ≤ 3 cm, confirming that in small nodules, morphological patterns such as the pleural tag—as conventional signs—are subtler or absent, and prediction becomes more challenging. In this scenario, our peritumoral models, particularly those derived from a 3 mm-peritumoral region, appeared to partially mitigate this limitation, maintaining a good sensitivity in nodules ≤ 3 cm compared to intratumoral models alone. This may have important clinical implications, as patients with small NSCLC are precisely those for whom the appropriateness of sublobar resections remains debated. The presence of an adverse prognostic factor such as VPI could therefore justify opting for lobectomy. Recent evidence suggests that VPI is an independent negative prognostic factor, even in tumors measuring ≤2–3 cm, affecting both recurrence and overall survival [27,28]. In this context, it is reasonable to expect that the future of personalized surgical decision-making in early-stage NSCLC will increasingly rely on an integrated approach combining clinical variables, radiomic features, and genomic information, consistent with recent studies highlighting the prognostic and predictive impact of molecular alterations in this setting.

### 4.1. Clinical Implications

Although exploratory and limited by its retrospective, single-center design, our study suggests that preoperative VPI prediction may provide clinically meaningful support in surgical planning for early-stage NSCLC. In particular, cases in which the predicted probability of VPI is high, particularly in tumors ≤ 3 cm or in part-solid nodules with a substantial solid component, may warrant consideration of lobectomy rather than anatomical segmentectomy, together with a more extensive lymph-node dissection or a lower threshold for conversion to thoracotomy if pleural or fissural involvement is suspected intraoperatively. Conversely, in settings where the model indicates a low probability of VPI, a parenchyma-sparing approach may be oncologically acceptable, helping to avoid unnecessary lobectomies and preserving postoperative lung function. The standard lymph-node evaluation would remain appropriate in these patients, and surgical judgment and intraoperative findings continue to play a central role.

These predictive probabilities may be valuable in borderline radiologic scenarios, such as ground-glass nodules with emerging solid components or small peripheral lesions near the pleura, where traditional CT morphologic signs are subtle, and decision-making is more subjective. Tumors ≤ 3 cm remain the subgroup in which improved VPI prediction may be most impactful, as even limited pleural invasion has been shown to affect prognosis in early-stage disease and may therefore influence the balance between limited and more extensive resection. In addition, in borderline stage IB tumors, identifying patients with a high predicted probability of VPI could help inform the selection of candidates who might benefit from adjuvant chemotherapy, while acknowledging that this consideration remains exploratory and requires prospective validation.

Because risk tolerance varies among surgeons, the clinical interpretation of predicted VPI probabilities will inevitably differ across institutions. Our results, therefore, should not be viewed as prescriptive but rather as hypothesis-generating, supporting the design of prospective radiomic-based studies aimed at defining and validating threshold probabilities.

### 4.2. Limitations of the Study

Our study presents some limitations, primarily related to its monocentric and retrospective nature, as well as its relatively small sample size, which may affect generalizability and underscore the need for larger validation studies. In particular, the absence of external validation is a key issue. Although internal robustness was assessed through stratified five-fold cross-validation and by estimating AUC confidence intervals via 1000 bootstrap replicates, these approaches cannot substitute for multi-center testing. Secondly, tumor segmentation, although carefully performed and reviewed by a single radiologist, remains subject to inter-observer variability. While we addressed the potential impact of manual segmentation variability through a perturbation-based robustness analysis in a patient’s subset—confirming that the features contributing to the final models were stable—this was an a posteriori assessment. In future studies, the robustness evaluation will be implemented as an active step in the feature selection workflow, ensuring that only stability-proven radiomic features are retained. Moreover, automation of the lesion delineation process—particularly through deep learning–based segmentation methods—would be desirable to further reduce variability and improve reproducibility.

Lastly, although all patients underwent FDG-PET for staging purposes, PET-derived metrics such as SUVmax were not included in the present analysis, as our primary aim was to characterize the predictive contribution of CT-based intratumoral and peritumoral radiomics. The integration of PET variables would require a multimodal modeling framework beyond the intended scope of this work. Nonetheless, given the recognized prognostic value of metabolic imaging [29,30], future studies are warranted to investigate whether PET metabolic parameters, in combination with CT radiomics, could further enhance VPI prediction.

## 5. Conclusions

In summary, our findings support the hypothesis that VPI is associated with detectable alterations in both tumoral and peritumoral microenvironment on contrast-enhanced CT. Incorporating radiomic features with clinical data enabled improved model performance compared to clinical-only models, yielding very good accuracies in both the training and validation cohorts. Even though our models are not yet ready for standalone clinical use, their performance is clinically informative and may become actionable once validated in larger prospective and multi-center studies.

## Figures and Tables

**Table 1:** Patient and tumor characteristics in the overall dataset, training set, and validation set.

Parameter	Overall *n* = 118	Training Set *n* = 82	Validation Set *n* = 36	*p*-Value
Gender				0.664
F	62 (53%)	42 (51%)	20 (56%)	
M	56 (47%)	40 (49%)	16 (44%)	
Smoking Status				0.714
active smoker	43 (36%)	29 (35%)	14 (39%)	
non-smoker	75 (64%)	53 (65%)	22 (61%)	
Age at Surgery (years)	69 (67, 70)	68.5 (66, 70)	69.5 (66, 72.5)	0.290
Major Axis Length (mm)	23.7 (19.9, 25.5)	23.7 (19.4, 25.7)	23.9 (17.7, 29.9)	0.928
Location				0.309
central	35 (30%)	22 (27%)	13 (36%)	
pheripheral	83 (70%)	60 (73%)	23 (64%)	
cTNM				0.999
1	82 (69%)	57 (70%)	25 (69%)	
2	26 (22%)	18 (22%)	8 (22%)	
3	10 (8.5%)	7 (8.5%)	3 (8.3%)	
Grading				0.373
2	55 (47%)	36 (44%)	19 (53%)	
3	63 (53%)	46 (56%)	17 (47%)	
Worst Histotype				
acinar	28 (24%)	22 (27%)	6 (17%)	
cribriform	5 (4.2%)	4 (4.9%)	1 (2.8%)	
micropapillary	12 (10%)	10 (12%)	2 (5.6%)	
papillary	37 (31%)	25 (30%)	12 (33%)	
solid	27 (23%)	15 (18%)	12 (33%)	
other	9 (7.6%)	6 (7.3%)	3 (8.3%)	
Spiculation				0.869
no	34 (29%)	24 (29%)	10 (28%)	
yes	84 (71%)	58 (71%)	26 (72%)	
Pleural Tag Sign				0.581
no	48 (41%)	32 (39%)	16 (44%)	
yes	70 (59%)	50 (61%)	20 (56%)	
Density				
solid	91 (78%)	62 (77%)	29 (83%)	
sub-solid	9 (7.8%)	7 (8.6%)	2 (5.7%)	
ground-glass	16 (14%)	12 (15%)	4 (11%)	
Surgery				0.507
left lower lobe	20 (17%)	16 (20%)	4 (11%)	
left upper lobe	24 (20%)	15 (18%)	9 (25%)	
right lower lobe	34 (29%)	26 (32%)	8 (22%)	
right middle lobe	5 (4.2%)	3 (3.7%)	2 (5.6%)	
right upper lobe	35 (30%)	22 (27%)	13 (36%)	
Visceral Pleural Invasion				0.862
no	80 (68%)	56 (68%)	24 (67%)	
yes	38 (32%)	26 (32%)	12 (33%)	

Numerical variables are reported as medians and 95% confidence intervals; categorical variables as counts. No *p*-value is reported for categorical variables with insufficient class counts. The Worst Histotype subtypes with low frequencies (clear-cell, lepidic, mixed, mucinous, pleomorphic, undifferentiated) are grouped under the category other. For the cTNM variable, stages 3 and 4 are merged into category 3. For the variable Grading, grades 1 and 2 are combined into category 2.

**Table 2:** Summary statistics of the best predictors included in the final models.

	No VPI	VPI	
Lesion Radiomic Predictor	Median [95% CI]	Median [95% CI]	*p*-Value
Integrated Intensity	2.0 [1.5, 2.8] × 10^6^	9.0 [4.8, 20.8] × 10^6^	<0.001
RMS Intensity	435 [401, 461]	370 [332, 393]	<0.001
Grey Level Non-Uniformity (GLSZM)	28 [25, 42]	79 [56, 229]	<0.001
**10 mm-rim Radiomic Predictor**	**Median [95% CI]**	**Median [95% CI]**	***p*****-Value**
Flatness	0.78 [0.75, 0.81]	0.68 [0.63, 0.72]	<0.001
Intensity Skewness	3.10 [2.85, 3.28]	2.40 [2.10, 2.54]	<0.001
Run Variance (GLRLM)	0.12 [0.11, 0.13]	0.09 [0.08, 0.10]	<0.001
	**No VPI**	**VPI**	
**Clinical Predictor**	**Counts**	**Counts**	***p*** **-Value**
Worst Histotype			
acinar	25	3	
cribriform	5	0	
micropapillary	4	8	
papillary	26	11	
solid	13	14	
other	7	2	
Pleural Tag Sign			<0.001
no	45	3	
yes	35	35	

The *p*-value for Worst Histotype is omitted due to insufficient class frequencies. The Worst Histotype subtypes with low frequencies (clear-cell, lepidic, mixed, mucinous, pleomorphic, undifferentiated) are grouped under the category other. Abbreviations: VPI, Visceral Pleural Invasion; CI, confidence interval; RMS, Root Mean Square; GLSZM, Grey-Level Size Zone Matrix; GLRLM, Grey-Level Run Length Matrix.

**Table 3:** Performance of the best–performing prediction models in the training and validation sets.

Model	Predictors	AUC	Accuracy	Sensitivity	Specificity	PPV	NPV
Clinical	Worst Histotype Pleural Tag Sign	0.81 [0.74, 0.88]	0.81 [0.73, 0.87]	0.91 [0.81, 0.96]	0.71 [0.59, 0.82]	0.76 [0.65, 0.85]	0.89 [0.77, 0.95]
		**0.83** **[0.68, 0.92]**	**0.81** **[0.65, 0.90]**	**0.92** **[0.65, 0.99]**	**0.75** **[0.55, 0.88]**	**0.65** **[0.41, 0.83]**	**0.95** **[0.75, 0.99]**
Radiomic_Lesion_	Integrated Intensity RMS Intensity Grey Level Non-Uniformity (GLSZM)	0.80 [0.72, 0.87]	0.80 [0.72, 0.87]	0.79 [0.66, 0.88]	0.82 [0.70, 0.90]	0.82 [0.69, 0.90]	0.79 [0.67, 0.88]
		**0.79** **[0.61, 0.91]**	**0.81** **[0.65, 0.90]**	**0.75** **[0.47, 0.91]**	**0.83** **[0.64, 0.93]**	**0.69** **[0.42, 0.87]**	**0.87** **[0.68, 0.96]**
Radiomic_10mm_	Flatness Intensity Skewness Run Variance (GLRLM)	0.83 [0.75, 0.89]	0.83 [0.75, 0.89]	0.84 [0.72, 0.91]	0.82 [0.70, 0.90]	0.83 [0.71, 0.90]	0.84 [0.72, 0.91]
		**0.79** **[0.60, 0.91]**	**0.81** **[0.65, 0.90]**	**0.75** **[0.47, 0.91]**	**0.83** **[0.64, 0.93]**	**0.69** **[0.42, 0.87]**	**0.87** **[0.68, 0.96]**
Clinical + Radiomic_Lesion_	RMS Intensity Worst Histotype Pleural Tag Sign	0.88 [0.80, 0.93]	0.88 [0.80, 0.92]	0.91 [0.81, 0.96]	0.84 [0.72, 0.91]	0.85 [0.74, 0.92]	0.90 [0.79, 0.96]
		**0.83** **[0.67, 0.94]**	**0.83** **[0.68, 0.92]**	**0.83** **[0.55, 0.95]**	**0.83** **[0.64, 0.93]**	**0.71** **[0.45, 0.88]**	**0.91** **[0.72, 0.98]**

Abbreviations: RMS, Root Mean Square; GLSZM, Grey-Level Size Zone Matrix; GLRLM, Grey-Level Run Length Matrix; AUC, area under the receiver operating characteristic curve; PPV, positive predictive value; NPV, negative predictive value. Bold values refer to the validation set. Performance metrics are reported with their corresponding 95% confidence intervals.

## Data Availability

All relevant data supporting the findings of this study are included in the article and its Supplementary Materials. Additional details are available from the corresponding author upon request.

## Supplementary Materials

The following supporting information can be downloaded at: https://www.mdpi.com/article/10.3390/cancers17244001/s1, Figure S1: Example of perturbation strategies (isotropic dilation/erosion and axial rotations) applied to the manual lesion segmentation of a representative patient with histologically confirmed lung adenocarcinoma; Figure S2: Bar plots illustrating the performance of the clinical model for predicting visceral pleural invasion across the three subgroups according to the lesion size; Figure S3: Bar plots illustrating the performance of the radiomic and combined models derived from the intratumoral volume for predicting visceral pleural invasion across the three subgroups based on the lesion size; Figure S4: Bar plots illustrating the performance of the radiomic and combined models derived from the 3 mm peritumoral volume for predicting visceral pleural invasion across the three subgroups based on the lesion size; Figure S5: Bar plots illustrating the performance of the radiomic and combined models derived from the 5 mm peritumoral volume for predicting visceral pleural invasion across the three subgroups based on the lesion size; Figure S6: Bar plots illustrating the performance of the radiomic and combined models derived from the 10 mm peritumoral volume for predicting visceral pleural invasion across the three subgroups based on the lesion size; Table S1: List of radiomic features included in the study; Table S2: Performance metrics of all the proposed models; Table S3: Intraclass Correlation Coefficients (ICCs) of radiomic features included in all developed models; Table S4: Comparison of model performances in the training and validation sets.

## Abbreviations

The following abbreviations are used in this manuscript:

VPI	Visceral Pleural Invasion
NSCLC	Non-Small Cell lung cancer
ML	Machine Learning
CT	Computed Tomography
LA	Lung Adenocarcinoma
AJCC	American Joint Committee on Cancer
UICC	Union for International Cancer Control
FDG-PET	F18-FluoroDeoxyGlucose Positron Emission Tomography
FFPE	Formalin-Fixed Paraffin-Embedded
H&E	Hematoxylin–Eosin
IASLC	International Association for the Study of Lung Cancer
WHO	World Health Organization
VOIs	Volumes of Interest
IBSI	Image Biomarker Standardization Initiative
GLCM	Grey-Level Co-occurrence Matrix
GLRLM	Grey-Level Run Length Matrix
NGTDM	Neighborhood Grey Tone Difference Matrix
GLSZM	Grey-Level Size Zone Matrix
AdaBoost	Adaptive Boosting
SMOTE-NC	Synthetic Minority Over-sampling Technique for Nominal and Continuous variables
AUC	Area Under the Curve
RMS	Root Mean Square
CI	Confidence Interval
PPV	Positive Predictive Value
NPV	Negative Predictive Value
