# Supplementary material for: Tumoral and Peritumoral Radiomics for Preoperative Prediction of Visceral Pleural Invasion in Lung Adenocarcinoma
Source: Cancers (Basel). 2025 Dec 16;17(24):4001. doi: 10.3390/cancers17244001 (PMC12731215; doi:10.3390/cancers17244001)
Supplement: Supplementary file 1 [file cancers-17-04001-s001.zip › cancers-3985654-supplementary.pdf]

cancers

MDPI

Supplementary Materials Content:

Tables

Table S1. List of radiomic features included in the study.

Table S2. Performance metrics of all the proposed models.

Table S3. Intraclass Correlation Coefficients (ICCs) of radiomic features included in all developed models.

Table S4. Comparison of model performances in the training and validation sets.

Figures

Figure S1. Example of perturbation strategies (isotropic dilation/erosion and axial rotations) applied to the manual lesion segmentation of a representative patient with histologically confirmed lung adenocarcinoma.

Figure S2. Bar plots illustrating the performance of the clinical model for predicting visceral pleural invasion across the three subgroups according to the lesion size.

Figure S3. Bar plots illustrating the performance of the radiomic and combined models derived from the intratumoral volume for predicting visceral pleural invasion across the three subgroups based on the lesion size.

Figure S4. Bar plots illustrating the performance of the radiomic and combined models derived from the 3 mm-peritumoral volume for predicting visceral pleural invasion across the three subgroups based on the lesion size.

Figure S5. Bar plots illustrating the performance of the radiomic and combined models derived from the 5 mm-peritumoral volume for predicting visceral pleural invasion across the three subgroups based on the lesion size.

Figure S6. Bar plots illustrating the performance of the radiomic and combined models derived from the 10 mm-peritumoral volume for predicting visceral pleural invasion across the three subgroups based on the lesion size.

Table S1. Complete list of the extracted radiomic features.

Feature Family	Number of Features	List of Features
**Morphological features**	17	Elongation *<br>Flatness *<br>Least Axis Length (mm) *<br>Major Axis Length (mm) *<br>Minor Axis Length (mm) *<br>Volume (cm^3)<br>Approximate Volume (cm^3)<br>Surface Area (cm^2)<br>Surface to Volume Ratio (cm)<br>Compactness 1

		Compactness 2<br>Spherical Disproportion<br>Sphericity<br>Asphericity<br>Centre f Mass Shift (mm)<br>Maximum 3D Diameter (mm)<br>Integrated Intensity
**Intensity-based features**	19	Mean Intensity<br>Intensity Variance<br>Intensity Skewness<br>Intensity Kurtosis<br>Median Intensity<br>Minimum Intensity<br>10th Intensity Percentile<br>50th Intensity Percentile<br>90th Intensity Percentile<br>Maximum Intensity<br>Intensity Interquartile Range<br>Intensity Range<br>Mean Absolute Deviation<br>Robust Mean Absolute Deviation<br>Median Absolute Deviation<br>Coefficient of Variation<br>Quartile Coefficient of Dispersion<br>Energy<br>Root Mean Square Intensity
**Intensity histogram features**	23	Mean<br>Variance<br>Skewness<br>Kurtosis<br>Median<br>Minimum Grey Level<br>10th Percentile<br>90th Percentile<br>Maximum Grey Level<br>Mode<br>Interquartile Range<br>Range<br>Mean Absolute Deviation<br>Robust Mean Absolute Deviation<br>Median Absolute Deviation<br>Coefficient of Variation<br>Quartile Coefficient of Dispersion<br>Entropy Log2<br>Uniformity<br>Maximum Histogram Gradient<br>Maximum Histogram Gradient Grey Level<br>Minimum Histogram Gradient<br>Minimum Histogram Gradient Grey Level

**GLCM – Grey Level Co-occurrence Matrix features**	23	Joint Maximum<br>Joint Average<br>Joint Variance<br>Joint Entropy Log2<br>Difference Average<br>Difference Variance<br>Difference Entropy<br>Sum Average<br>Sum Variance<br>Sum Entropy<br>Angular Second Moment<br>Contrast<br>Dissimilarity<br>Inverse Difference<br>Normalized Inverse Difference<br>Inverse Difference Moment<br>Normalized Inverse Difference Moment Inverse Variance<br>Correlation<br>Autocorrelation<br>Cluster Tendency<br>Cluster Shade<br>Cluster Prominence
**GLRLM – Grey Level Run Length Matrix features**	16	Grey Level Non Uniformity Normalized *<br>Grey Level Variance *<br>Run Entropy *<br>Run Length Non Uniformity Normalized *<br>Run Variance *<br>Short Runs Emphasis<br>Long Runs Emphasis<br>Low Grey Level Run Emphasis<br>High Grey Level Run Emphasis<br>Short Run Low Grey Level Emphasis<br>Short Run High Grey Level Emphasis<br>Long Run Low Grey Level Emphasis<br>Long Run High Grey Level Emphasis<br>Grey Level Non Uniformity<br>Run Length Non Uniformity<br>Run Percentage
**NGTDM – Neighborhood Grey Tone Difference Matrix features**	5	Coarseness<br>Contrast<br>Busyness<br>Complexity<br>Strength
**GLSZM – Grey Level Size Zone Matrix features**	16	Small Zone Emphasis<br>Large Zone Emphasis<br>Low Grey Level Zone Emphasis<br>High Grey Level Zone Emphasis<br>Small Zone Low Grey Level Emphasis<br>Small Zone High Grey Level Emphasis

	Large Zone Low Grey Level Emphasis
	Large Zone High Grey Level Emphasis
	Grey Level Non-Uniformity
	Normalized Grey Level Non-Uniformity
	Zone Size Non-Uniformity
	Normalized Zone Size Non-Uniformity
	Zone Percentage
	Grey Level Variance
	Zone Size Variance
	Zone Size Entropy

* Features extracted using the PyRadiomics library (version 3.0.1).

Table S2. Summary of performance metrics across all developed models.

Model	Accuracy	Sensitivity	Specificity
	0.81	0.91	0.71
**Clinical**	[0.73, 0.87]	[0.81, 0.96]	[0.59, 0.82]
1) Worst Histotype			
2) Pleural Tag Sign	**0.81**	**0.92**	**0.75**
	**[0.65, 0.90]**	**[0.65, 0.99]**	**[0.55, 0.88]**
**Radiomic**	0.80	0.79	0.82
**(Lesion)**	[0.72, 0.87]	[0.66, 0.88]	[0.70, 0.90]
1) Integrated Intensity			
2) RMS Intensity	**0.81**	**0.75**	**0.83**
3) Grey Level Non-Uniformity (GLSZM)	**[0.65, 0.90]**	**[0.47, 0.91]**	**[0.64, 0.93]**
**Radiomic**	0.81	0.86	0.77
**(3 mm-peritumoral)**	[0.73, 0.87]	[0.74, 0.93]	[0.64, 0.86]
1) Minor Axis Length			
2) Intensity Skewness	**0.81**	**0.83**	**0.79**
3) RMS Intensity	**[0.65, 0.90]**	**[0.55, 0.95]**	**[0.60, 0.91]**
**Radiomic**	0.72	0.79	0.66
**(5 mm-peritumoral)**	[0.63, 0.81]	[0.67, 0.89]	[0.53, 0.79]
1) Compactness 1			
2) Long Runs Emphasis (GLRLM)	**0.78**	**0.75**	**0.80**
3) Quartile Coefficient of Dispersion	**[0.64, 0.92]**	**[0.49, 0.99]**	**[0.63, 0.96]**
**Radiomic**	0.83	0.84	0.82
**(10 mm-peritumoral)**	[0.75, 0.89]	[0.72, 0.91]	[0.70, 0.90]
1) Flatness			
2) Intensity Skewness	**0.81**	**0.75**	**0.83**
3) Run Variance (GLRLM)	**[0.65, 0.90]**	**[0.47, 0.91]**	**[0.64, 0.93]**
**Combined**	0.88	0.91	0.84
**(Lesion)**	[0.80, 0.92]	[0.81, 0.96]	[0.72, 0.91]
1) RMS Intensity			
2) Worst Histotype	**0.83**	**0.83**	**0.83**
3) Pleural Tag Sign	**[0.68, 0.92]**	**[0.55, 0.95]**	**[0.64, 0.93]**
**Combined**	0.86	0.91	0.80
**(clinical + 3 mm-peritumoral)**	[0.78, 0.91]	[0.81, 0.96]	[0.68, 0.89]
1) Intensity Skewness			
2) Worst Histotype	**0.83**	**0.83**	**0.83**
3) Pleural Tag Sign	**[0.68, 0.92]**	**[0.55, 0.95]**	**[0.64, 0.93]**

**Combined (clinical + 5 mm-peritumoral)**<br>1) Compactness 1<br>2) Worst Histotype<br>3) Pleural Tag Sign	0.81<br>[0.73, 0.87]<br>**0.83**<br>**[0.68, 0.92]**	0.89<br>[0.79, 0.95]<br>**0.92**<br>**[0.65, 0.99]**	0.73<br>[0.60, 0.83]<br>**0.79**<br>**[0.60, 0.91]**
**Combined (clinical + 10 mm-peritumoral)**<br>1) Low Grey Level Zone Emphasis (GLSZM)<br>2) Worst Histotype<br>3) Pleural Tag Sign	0.86<br>[0.78, 0.91]<br>**0.83**<br>**[0.68, 0.92]**	0.91<br>[0.81, 0.96]<br>**0.83**<br>**[0.55, 0.95]**	0.80<br>[0.68, 0.89]<br>**0.83**<br>**[0.64, 0.93]**

Abbreviations: RMS, Root Mean Square; GLSZM, Grey-Level Size Zone Matrix; GLRLM, Grey-Level Run Length Matrix. **Bold** values refer to the validation set. Performance metrics are reported with their corresponding 95% confidence intervals.

Table S3. Intraclass Correlation Coefficients (ICCs) of selected radiomic features across all developed models.

Model	ICC [95% CI]
**Radiomic (Lesion)**	
- Integrated Intensity	0.97 [0.95, 0.99]
- RMS Intensity	0.88 [0.79, 0.94]
- Grey Level Non-Uniformity (GLSZM)	0.94 [0.90, 0.97]
**Radiomic (3 mm-peritumoral)**	
- Minor Axis Length	0.99 [0.99, 1]
- Intensity Skewness	0.85 [0.75, 0.93]
- RMS Intensity	0.88 [0.79, 0.95]
**Radiomic (5 mm-peritumoral)**	
- Compactness 1	0.96 [0.92, 0.98]
- Long Runs Emphasis (GLRLM)	0.94 [0.89, 0.97]
- Quartile Coefficient of Dispersion	0.93 [0.87, 0.97]
**Radiomic (10 mm-peritumoral)**	
- Flatness	0.98 [0.96, 0.99]
- Intensity Skewness	0.97 [0.94, 0.99]
- Run Variance (GLRLM)	0.98 [0.97, 0.99]
**Combined (clinical +10 mm-peritumoral)**	
- Low Grey Level Zone Emphasis (GLSZM)	0.99 [0.98, 1]

Abbreviations: ICC, Intraclass Correlation Coefficient; CI, confidence interval; RMS, Root Mean Square; GLSZM, Grey-Level Size Zone Matrix; GLRLM, Grey-Level Run Length Matrix.

Table S4. Comparison of Model Performances in the Training and Validation Sets, with p-values from the McNemar's Test.

Training Set

Model	Clinical	Radiomic (Lesion)	Radiomic (10 mm)	Combined
**Clinical**	1.00	0.86	0.72	0.008
**Radiomic (Lesion)**		1.00	0.58	0.12
**Radiomic (10 mm)**			1.00	0.33
**Combined**				1.00

	Validation Set			
**Model**	**Clinical**	**Radiomic (Lesion)**	**Radiomic (10 mm)**	**Combined**
**Clinical**	1.00	1.00	1.00	0.63
**Radiomic (Lesion)**		1.00	1.00	0.75
**Radiomic (10 mm)**			1.00	0.75
**Combined**				1.00

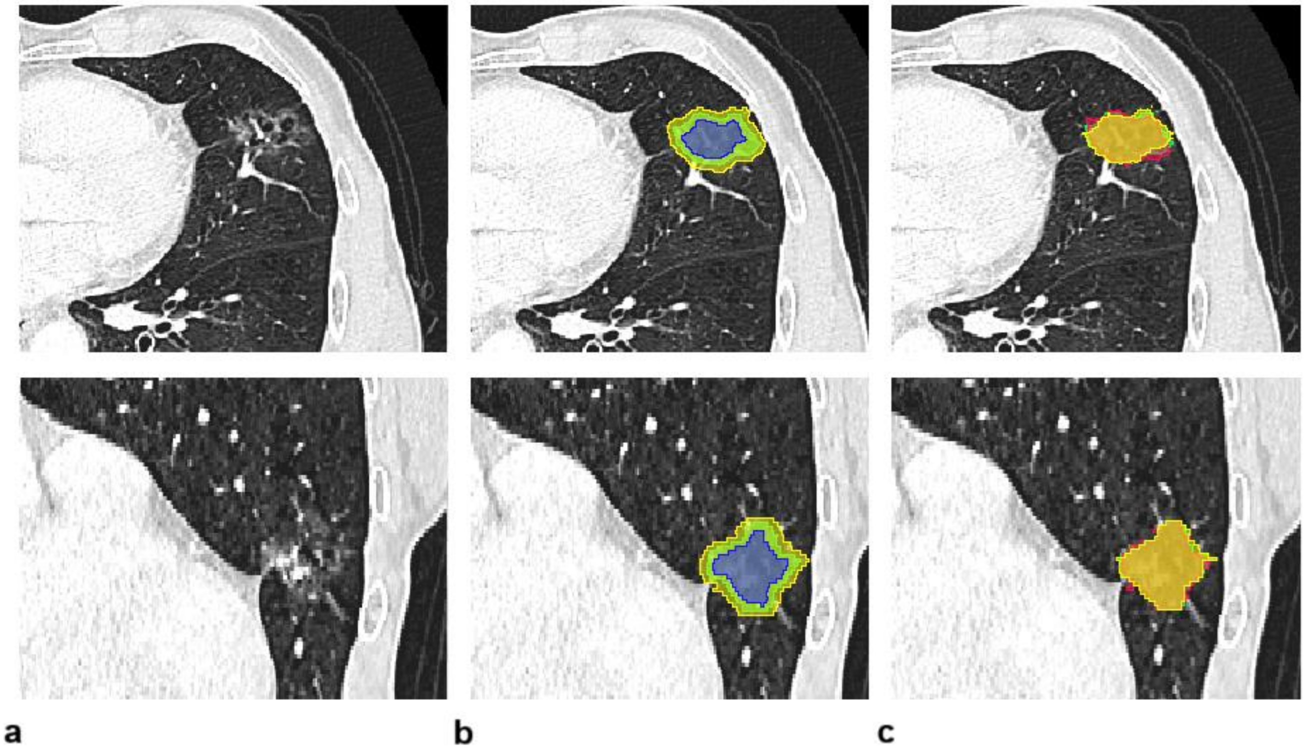

Figure S1. Representative axial and coronal CT images of a patient with histologically confirmed lung adenocarcinoma. The first column shows the reference images (a). In the second column (b), the original lesion segmentation (green) is compared with masks generated by isotropic dilation of 2 mm (yellow) and isotropic erosion of 2 mm (blue). In the third column (c), the original lesion segmentation (green) is compared with masks generated by applying ±10° rotations (red and yellow, respectively) around the axial plane to the original mask.

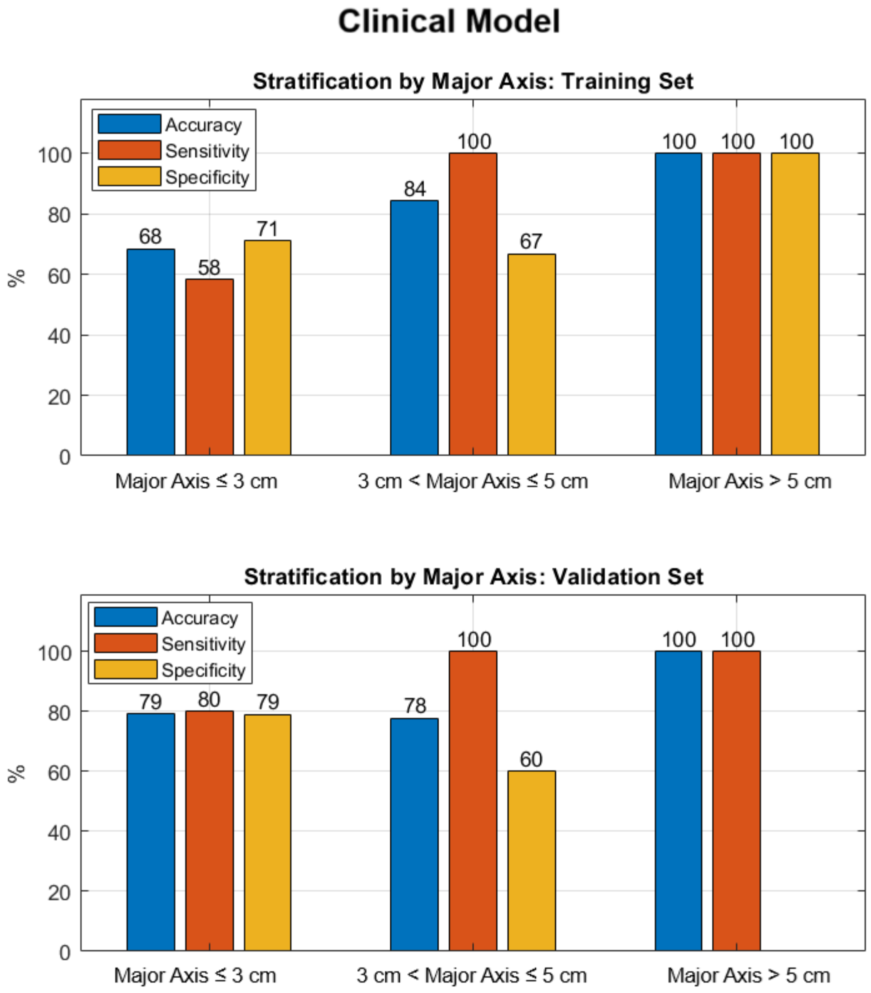

Figure S2. Bar plots illustrating the performance of the clinical model for predicting visceral pleural invasion across the three subgroups according to the lesion size.

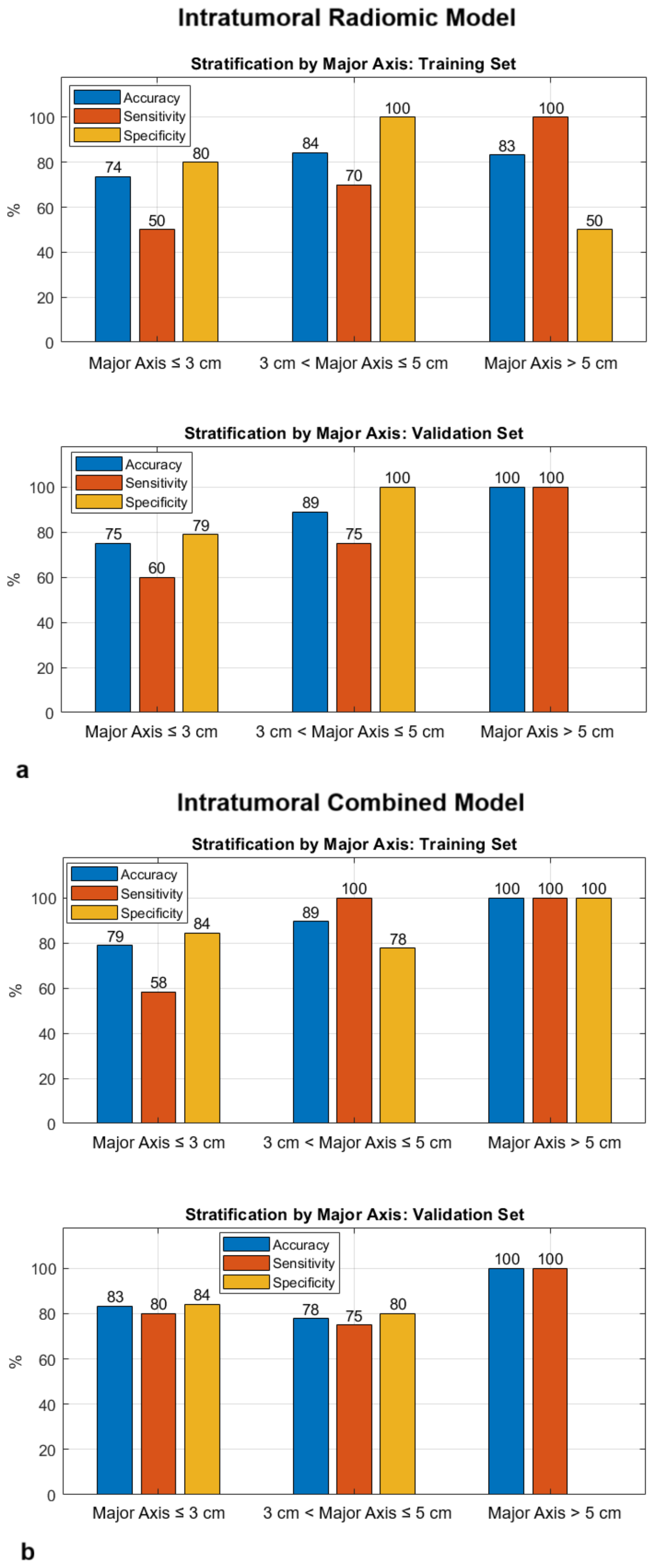

Figure S3. Bar plots illustrating the performance of the radiomic (a) and combined (b) models derived from the intratumoral volume for predicting visceral pleural invasion across the three subgroups based on the lesion size.

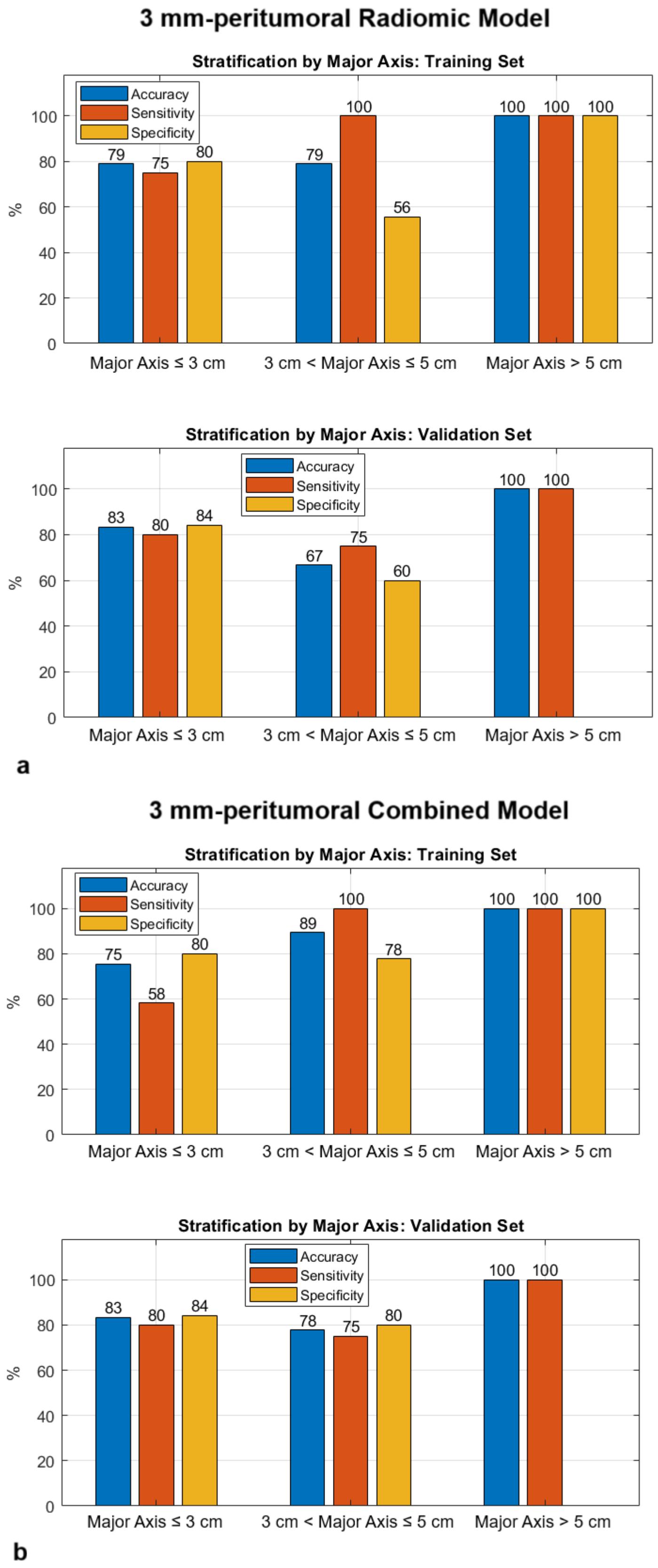

Figure S4. Bar plots illustrating the performance of the radiomic and combined models derived from the 3 mm-peritumoral volume for predicting visceral pleural invasion across the three subgroups based on the lesion size.

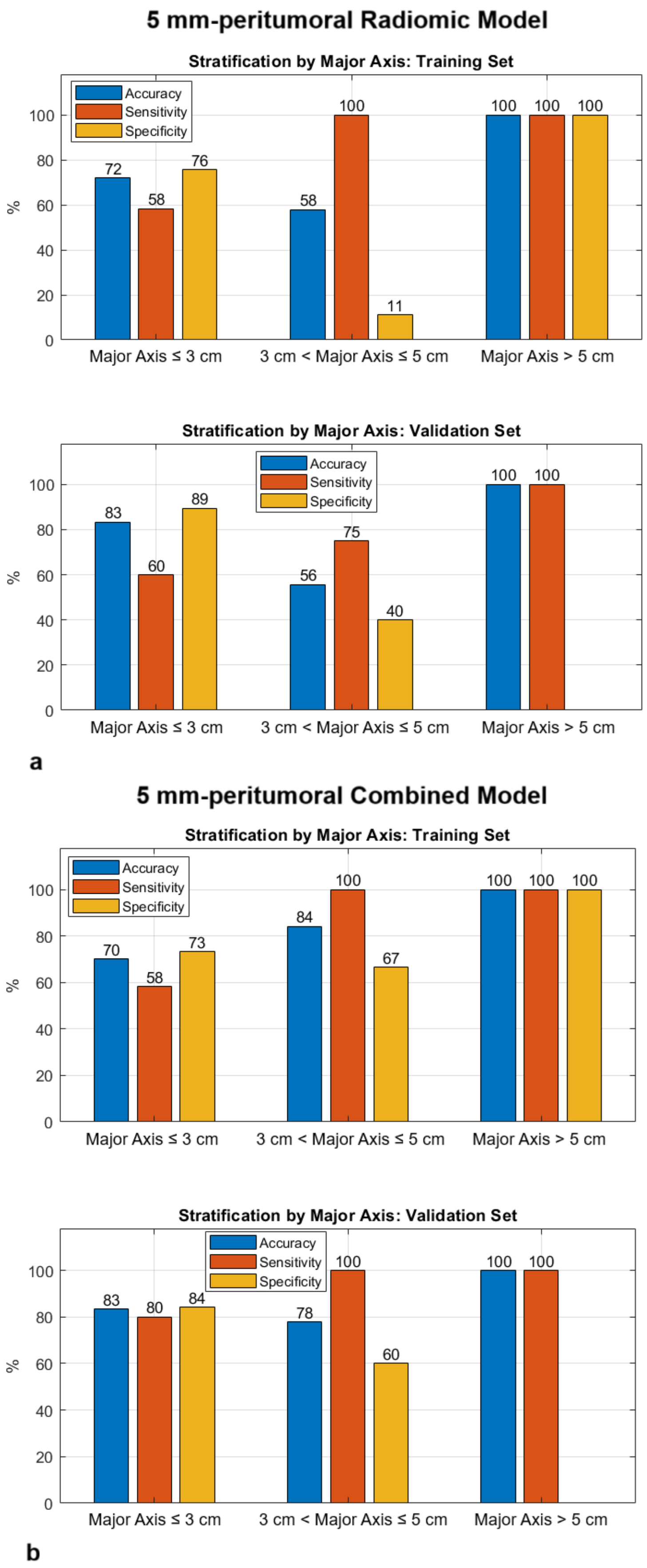

Figure S5. Bar plots illustrating the performance of the radiomic and combined models derived from the 5 mm-peritumoral volume for predicting visceral pleural invasion across the three subgroups based on the lesion size.

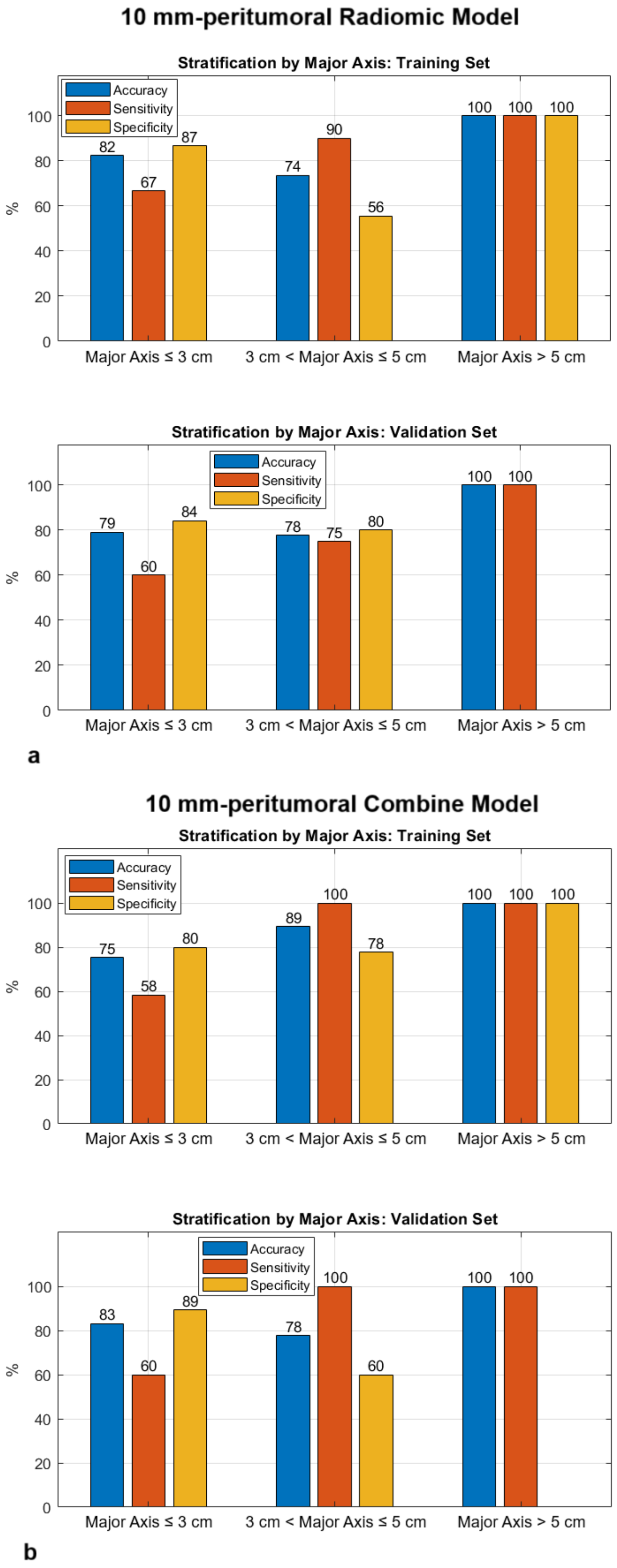

Figure S6. Bar plots illustrating the performance of the radiomic and combined models derived from the 10 mm-peritumoral volume for predicting visceral pleural invasion across the three subgroups based on the lesion size.
